# Risk factors analysis of attention deficit/hyperactivity disorder and allergic rhinitis in children: a cross-sectional study

**DOI:** 10.1186/s13052-019-0703-1

**Published:** 2019-08-13

**Authors:** Keyang Chen, Xiuxiu Zheng, Ziyue Li, Haijie Xiang, Bobei Chen, Hui Zhang

**Affiliations:** 1 Department of Neurology, The Second Affiliated Hospital and Yuying Children’s Hospital of Wenzhou Medical University, Wenzhou, Zhejiang China; 2 Department of Otolaryngology, The Second Affiliated Hospital and Yuying Children’s Hospital of Wenzhou Medical University, College West Road No. 109, Wenzhou, 325027 Zhejiang China; 3 Department of Pediatric Allergy and Immunology, The Second Affiliated Hospital and Yuying Children’s Hospital of Wenzhou Medical University, College West Road No. 109, Wenzhou, 325027 Zhejiang China

**Keywords:** Rhinitis, Allergic, Attention-deficit/hyperactivity disorder, Pediatric

## Abstract

**Background:**

To investigate the relationship between symptom of attention-deficit/hyperactivity disorder (ADHD) and allergic rhinitis (AR) in AR children of different genders and ages.

**Methods:**

Four hundred and sixty-five allergic rhinitis children aged 6–12 years old were recruited in this study. Skin-prick test, Pediatric Rhinoconjunctivitis Quality of Life Questionnaire (PRQLQ), Total Nasal Symptoms Score (TNSS) and the Swanson, Nolan, and Pelham version IV scale (SNAP-IV) were recorded. Patients were divided into AR with ADHD and AR without ADHD, according to the SNAP-IV scale results.

**Results:**

Children with the inattention/hyperactivity scale (IHS) > 1.25 accounted for 26.4% of all children with AR. The TNSS with IHS > 1.25 group were significantly higher than the IHS ≤ 1.25 group. Univariate analysis showed that age, gender, duration of AR symptoms, skin index, and PRQLQ subscales were associated with symptoms of hyperactivity and attention deficit (IHS > 1.25). After normalizing the age and gender factors, duration of AR symptoms and skin index correlated with IHS > 1.25. After stratifying age and gender, the correlation between IHS > 1.25 and skin index and PRQLQ subscales was mainly found in male children, and the association between the duration of AR symptoms and IHS > 1.25 was reflected in each group.

**Conclusions:**

ADHD in children with AR is associated with severity, duration, and skin index of AR, and this association is more pronounced in male children.

**Electronic supplementary material:**

The online version of this article (10.1186/s13052-019-0703-1) contains supplementary material, which is available to authorized users.

## Background

Attention-deficit/hyperactivity disorder (ADHD) is one of the most frequently diagnosed disorder found in both children and adults. ADHD is manifested by inattention, hyperactivity, cognitive deficit, and/or impulsivity. It affects approximately 3–5% of youth and 2.5–5% of adults [1]. The incidence of ADHD in China is 6.26% [2]; 9.5% among individuals aged 4 to 17 years old in the United States [3] and 7.2% worldwide [4]. ADHD-related neuropsychological deficits affect academic, social, professional functioning, and impose significant economic burdens on the society [5]. ADHD is associated with many diseases, including cognitive impairment, sleep disorders and allergic diseases. Allergic rhinitis (AR) is one of the most common allergic diseases affecting children. Prevalence of global childhood allergic rhinitis has been reported to be as high as 40% [6]. Allergic rhinitis and ADHD affect children of similar ages [7]. Symptoms of allergic rhinitis may lead to daytime inattention, irritability and hyperactivity, which is commonly observed in ADHD children. Although there have been studies showing that AR has a strong relationship with ADHD [8, 9], the mechanism is still controversial. The prevalence of ADHD and allergic disease has increased worldwide. Both allergy and ADHD rely on gene-environment interaction. At present, there are few studies in this area in China. Our previous research has confirmed that children with AR have higher ADHD-related symptom scores than children without AR [10]. In this study, we therefore investigated the association between the symptom of AR and ADHD in children with AR at different age and gender groups.

## Methods

### Study design and setting

A cross-sectional study was performed to investigate the relationship between symptom of ADHD and AR in AR children of different genders and ages at the Second Affiliated Hospital of Wenzhou Medical University (Yuying Children’s Hospital), China. All the parents and/or participants signed informed consent forms before the assessment and were evaluated by a pediatrician/otolaryngologist and neurologist. The study was approved by the Second Affiliated Hospital of Wenzhou Medical University (Yuying Children’s Hospital) Ethics Committee.

### Study participants and data source

The patients were recruited in the ear, nose, and throat/pediatrician outpatient clinic over a 2-year period from September 2016 to December 2018. Children with AR were enrolled based on the Allergic Rhinitis and its Impact on Asthma (ARIA) guidelines [11, 12]. An ADHD diagnosis is dependent on clinical observations, rating scales from multiple informants (e.g, teachers and parents), and documentation of clinically significant impairment [13]. Inclusion criteria are: (a) age between the 6 and 12 years old; (b) children have 2 of 4 basic symptoms for, more than 1 h; (c) The skin prick test (SPT) has clear allergens. Exclusion criteria are: (a) children with ADHD undergoing medical treatment; (b) children with mental retardation or growth retardation; (c) children with other mental illnesses (such as depression and epilepsy); (d) Child/family does not cooperate during research.

### AR symptom scores

The Total Nasal Symptoms Score (TNSS), used to assessing nasal symptoms, is the sum of the scores of the four nasal symptoms (watery rhinorrhea, sneezing, nasal obstruction, and nasal pruritus) on a scale of 0 to 3 (0, none; 1, mild; 2, moderate; and 3, severe).

### Skin-prick tests

Skin-prick tests were performed by using 18 common Chinese inhaled and food allergens as well as negative and positive controls. The allergens tested were those of dermatophagoides pteronyssinus, dermatophagoides farinae, cockroach, the molds alternaria alternate and aspergillus fumigatus, Baker’s yeast, wheat, sieversiana pollen, sesame, croaker, crab, silk, shrimp, cat, dog, egg, milk, soybean, and peanut. A mean wheal diameter of 3 mm that of the negative control was considered positive. Atopy was considered to be present when one or more allergens yielded a positive skin-prick test result. To determine the reaction intensity of SPT, we can calculate the skin index (SI), namely the ratio of food sensation-induced wheal to histamine control response, which can reveal differences in individual skin responsiveness [14] where + is SI ≤ 0.5; ++ is 0.5 < SI ≤ 1; +++ is 1 < SI ≤ 2; ++++is SI > 2 [15].

### Pediatric Rhinoconjunctivitis quality of life questionnaire (PRQLQ)

The Pediatric Rhinoconjunctivitis Quality of Life Questionnaire [16] consists of 23 questions, which cover five aspects: nose symptoms, eye symptoms, practical problems, other symptoms, and activity limitations. The score of each question ranges from 0 to 6. A score of 0 indicates no impairment. The children were asked to score their past 7 days of experiences. Validation of the standardized version of the PRQLQ showed it to have satisfactory levels of reliability and concurrent validity [17].

### The Swanson, Nolan, and Pelham version IV scale (SNAP-IV)

The Swanson, Nolan, and Pelham version IV scale (SNAP-IV) is a 26-item scale used to evaluate ADHD symptoms and its severity [18]. It includes 18 questions on ADHD symptoms (9 on inattention and 9 on hyperactivity/impulsivity) and 8 questions on oppositional defiant disorder symptoms. Each item is scored on a scale of 0 (not at all) to 3 (very much). The Chinese version of the SNAP-IV has been used to yield reliable and valid results [19].

### The inattention/hyperactivity scale (IHS)

The inattention/hyperactivity scale (IHS) asks parents to rate their children for 18 DSM-IV category A symptoms of ADHD (Additional file 1: Table S1), which has a long history and the validity of the instrument [20]. In the DSM-IV, 6 chronic symptoms among 9 that relate to inattention, plus 6 among 9 that relate to hyperactive/impulsive behavior, are required to support a diagnosis of ADHD, combined type. Judgement provided on a four-point Likert scale from 0 to 3; a mean item response (IHS score) higher than 1.25 is considered to have symptoms of hyperactivity and attention deficit. This threshold corresponds to 12 or more positive responses (2, applies quite a bit or 3, definitely applies most of the time) among the 18 symptoms-items [21].

### Statistical analysis

Data was collected and statistically analyzed using SPSS version 22. Comparison of data was done using Student’s t-test for parametric data and chi-square χ2 test for non-parametric data. Two tailed *p* value of *p* < 0.05 was regarded as significant. Binary logistic regression analysis of association between AR-related symptoms and IHS children. Odds ratios with 95% confidence intervals (95% CIs) were calculated by logistic regression. The relative OR values of all independent variables and their 95% confidence intervals control age and gender factors. At the same time, the age and gender were stratified and the above analysis was repeated.

## Results

### Clinical observations

**Fig. 1:**
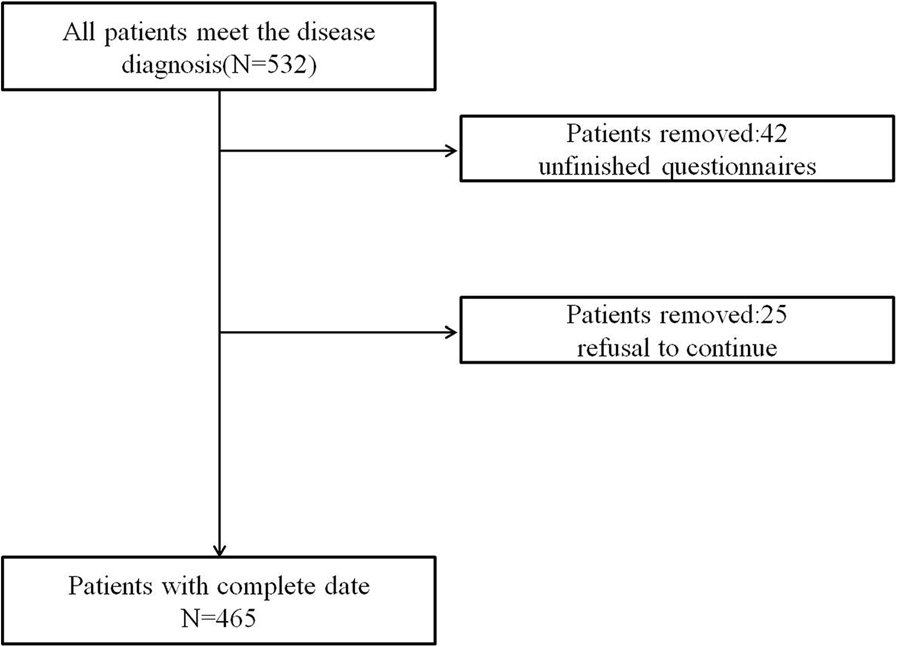
The flow chart of sample inclusion and exclusion

532 participants were recruited for this study. Patients with unfinished questionnaires or refusal to continue (*n* = 67) were excluded. Therefore, 465 participants (218 male and 247 female) were analyzed in the current study (Fig. 1) with the average age (9.52 ± 1.60) years old. Patients were divided into IHS > 1.25 group (122 cases, 26.4%) and IHS ≤ 1.25 group (343 cases). The nasal symptom scores and PRQLQ scores of the IHS > 1.25 group were higher than the IHS ≤ 1.25 group, as shown in Table 1. SPT results showed that most of the inhalant allergen was Dermatophagoides pteronyssinus, and the most common food allergen was shrimp (Table 2).

**Table 1:** Clinical data of IHS > 1.25 group and IHS ≤ 1.25 group

variable	Total (*n* = 465)	IHS > 1.25 group (*n* = 122)	IHS ≤ 1.25 group (*n* = 343)	*P*
Age	9.53 ± 1.60	8.59 ± 1.41	9.79 ± 1.48	< 0.01
gender	219/246	68/54	151/192	< 0.01
Other allergic diseases (merged/not combined)	121/344	35/87	86/257	0.423
Allergens (simple/mixed)	302/163	74/48	228/115	0.221
Family history (with/without)	138/327	35/87	103/240	0.805
TNSS				
SNAP-IV	5.68 ± 2.00	7.38 ± 1.69	5.04 ± 1.79	< 0.01
Inattention	9.70 ± 5.40	15.48 ± 4.29	7.61 ± 4.01	< 0.01
Hyperactivity/impulsivity	8.51 ± 5.03	13.91 ± 2.77	6.49 ± 3.13	< 0.01
IHS	1.01 ± 0.47	1.68 ± 0.29	0.78 ± 0.39	< 0.01
PRQLQ				
Nasal symptom score	2.46 ± 1.28	3.55 ± 1.09	2.04 ± 1.21	< 0.01
Ocular symptoms score	1.79 ± 0.99	2.34 ± 0.96	1.67 ± 0.93	< 0.01
Behavioral problem score	1.99 ± 0.88	2.61 ± 0.81	1.77 ± 0.84	< 0.01
Non-eye and nose symptoms score	1.34 ± 0.95	2.04 ± 0.83	1.09 ± 0.84	< 0.01
Activity and sleep score	1.25 ± 0.77	1.74 ± 0.75	1.06 ± 0.73	< 0.01
AR duration in 1 year				
≤ 1 month	142(30.5)	11 (8.6)	131 (38.1)	< 0.01
1~3 months	139(29.9)	22 (18.1)	117 (34.1)	< 0.01
3~6 months	112(24.1)	57 (46.6)	55 (16.1)	< 0.01
6~9 months	49(10.5)	24 (19.8)	25 (7.4)	< 0.01
9~12 months	23(4.9)	8 (6.9)	15 (4.3)	0.267
Skin index				
+	124(26.7)	17 (13.8)	107 (31.3)	< 0.01
++	182(39.1)	47 (38.8)	135 (39.3)	0.948
+++	138(29.7)	48 (39.7)	90 (26.3)	< 0.01
++++	21(4.5)	10 (7.8)	11 (3.1)	0.031

*P:comparison between IHS > 1.25 group and IHS ≤ 1.25 group*

**Table 2:** Results of SPT for each allergen in children

	Number of positive case	Sensitizationrate /%	Allergen distribution/%
Inhalation allergen			
Dermatophagoidespteronyssinus	322	69.2	24.4
Dermatophagoidesfarinae	310	66.7	23.5
cockroach	63	13.5	4.8
the molds Alternaria alternate and Aspergillus fumigatus	8	1.7	0.6
baker’s yeast	41	8.8	3.1
crab	83	17.8	6.3
silk	43	9.2	3.3
Sieversiana pollen	68	14.6	5.2
cat	54	11.6	4.1
dog	64	13.8	4.9
Food allergen			
sesame	42	9.0	3.2
croaker	29	6.2	2.2
wheat	8	1.7	0.6
shrimp	84	18.1	6.4
egg	25	5.4	1.9
milk	39	8.4	2.9
soybean	14	3.0	1.1
peanut	21	4.5	1.6

### Univariate analysis and binary logistic regression results

Univariate analysis was performed on relevant risk factors listed in Table 3. The analysis showed that family history (*P* = 0.792), allergen type (*P* = 0.276), and other allergic diseases (*P* = 0.413) had no correlation with attention deficit and hyperactivity symptoms. However, the skin index and symptom presence time of the child are risk factors for attention deficit and hyperactivity symptoms after normalizing gender and age factors (Table 4). In addition, multiple subscales of the PRQLQ are also significantly correlated with IHS > 1.25.

**Table 3:** Specific factors of relevant factors

Factor	Assignment description
gender	Male = 1, female = 0
Age	6 years old = 1, 7 years old = 2 … .12 years old = 7
Family history	Yes = 1, No = 0
AR duration in 1 year	≤1 month = 1, 1~3 months = 2, 3~6 months = 3, 6~9 months = 4, 9~12 months = 5
Skin index	+ = 1, ++ = 2, +++ = 3, ++++ = 4
Allergen species	Simple = 0, mixed = 1
Other allergic diseases	No merge = 0, merge =1
Nasal symptom score	< 1 = 1,1- = 2,2- = 3, … 5- = 6
Ocular symptoms score	< 1 = 1,1- = 2,2- = 3, … 5- = 6
Behavioral problem score	< 1 = 1,1- = 2,2- = 3, … 5- = 6
Non-eye and nose symptoms score	< 1 = 1,1- = 2,2- = 3, … 5- = 6
Activity and sleep score	< 1 = 1,1- = 2,2- = 3, … 5- = 6

**Table 4:** Results of binary logistic regression analysis of AR-related symptoms for IHS > 1.25

Factor	β	OR	95% CI	*P*
AR duration in 1 year	0.589	1.807	1.353~2.418	< 0.01
Skin index	0.651	1.912	1.321~2.771	< 0.01
Nasal symptom score	0.507	1.657	1.181~2.324	< 0.01
Ocular symptoms score	0.283	1.324	0.929~1.890	0.112
Behavioral problem score	0.911	2.483	1.761~3.501	< 0.01
Non-eye and nose symptoms score	0.351	1.418	0.915~2.191	0.119
Activity and sleep score	0.717	2.045	1.398~2.991	< 0.01

Control age and gender factors

### Post-stratification analysis of age and gender

In 218 male patients, IHS has significant correlation with symptom duration, skin index, and PRQLQ partial subscale. In 247 female patients, IHS > 1.25 was only associated with duration of symptoms and 1 component (Rhinitis behavior problem score). The relationship between skin index and IHS > 1.25 was not statistically significant (*P* = 0.108) (Table 5).

**Table 5:** Results of binary logistic regression analysis of AR-related symptoms for IHS > 1.25

Factor	Male				Female			
	β	OR	95% CI	*P*	β	OR	95% CI	*P*
AR duration in 1 year	0.438	1.551	1.057~2.274	0.025	0.941	2.563	1.523~4.320	< 0.01
Skin index	0.776	2.171	1.349~3.496	< 0.01	0.538	1.714	0.887~3.317	0.108
Nasal symptom score	0.530	1.701	1.084~2.663	0.022	0.281	1.326	0.756~2.326	0.327
Ocular symptoms score	0.275	1.316	0.836~2.073	0.234	0.353	1.423	0.746~2.714	0.285
Behavioral problem score	1.057	2.879	1.834~4.522	< 0.01	0.582	1.789	0.968~3.305	0.064
Non-eye and nose symptoms score	0.042	1.047	0.603~1.809	0.878	1.121	3.064	1.329~7.075	< 0.01
Activity and sleep score	0.720	2.055	1.281~3.296	< 0.01	0.550	1.733	0.830~3.620	0.143

Control age factors

Skin index and duration of symptoms were significantly associated with IHS > 1.25 in groups aged ≤8 years and > 8 years. Logistic regression analysis showed no significant association between gender and age and skin index, 1-year symptom duration, and PRQLQ scale (Table 6).

**Table 6:** Results of binary logistic regression analysis of AR-related symptoms for IHS > 1.25

Factor	≤8 years old				> 8 years old			
	β	OR	95% CI	*P*	β	OR	95% CI	*P*
AR duration in 1 year	0.565	1.758	1.068~2.887	0.03	0.716	2.047	1.379~3.035	< 0.01
Skin index	0.668	1.949	1.033~3.671	0.04	0.716	2.046	1.233~3.391	< 0.01
Nasal symptom score	0.662	1.938	1.136~3.307	0.015	0.304	1.356	0.812~2.265	0.245
Ocular symptoms score	0.267	1.303	0.742~2.382	0.388	0.277	1.319	0.815~2.135	0.260
Behavioral problem score	0.642	1.902	1.049~3.446	0.035	1.113	3.042	1.897~4.877	< 0.01
Non-eye and nose symptoms score	0.136	1.147	0.518~2.538	0.737	0.631	1.878	1.046~3.378	0.036
Activity and sleep score	1.091	2.974	1.474~6.001	< 0.01	0.456	1.577	0.968~2.565	0.067

Control age and gender factors

## Discussion

The association between ADHD and allergic diseases has been a source of public and clinical concern. The epidemiological research suggests that allergic diseases may increase the risk of ADHD in children, especially allergic rhinitis [22]. Treatment for AR may have a positive effect on behavior [23]. Children with ADHD and allergic disease may have a common biological background [24]. A number of studies have suggested a link between allergies and ADHD, such as food allergies and neuropsychiatric conditions [25] or immune disorder [26].

ADHD symptoms among children with allergic diseases including allergic rhinitis have been reported [27]. Conversely, allergic symptoms in children with ADHD have also been reported [28]. In our cross-sectional study, we demonstrated that AR with ADHD had more severe nasal symptoms than children without ADHD, in accordance with other research [29]. The single factor analysis found that there is no significant correlation between the number of allergens and the symptoms of ADHD, which was similar to a population-based case-control study [30]. The study showed that the association between allergic diseases and ADHD was occurred mostly due to house dust mites only, but not in other types of allergens. Further evaluation and follow-up study are needed for the conclusion. In addition, our study further showed that family history and whether or not combined with other allergic diseases were not significantly associated with ADHD.

Based on these results, we suggest that there is some overlap in the mechanism of action of allergy and ADHD in patients with a comorbid diagnosis. This study also found that the proportion of male patients is significantly higher than that of female children, and the average age of children is younger in IHS > 1.25 group, which suggests that young male children have more severe symptoms associated with ADHD group. Some previous studies in the United States have also found the same trend [3, 31]. To more accurately investigate the association of AR-related factors with ADHD symptoms, we control age and gender factors. Based on the Logistics regression analysis results, we observed that the severity of attention deficits and hyperactivity were significantly associated with multiple factors in AR children. AR children with attention deficit and hyperactivity have a longer duration of symptoms in 1 year compared with AR children without ADHD. This phenomenon exists in different age and gender groups. In addition, we also identified a positive association between ADHD and AR by positive SPT as Yang described [30]. However, in our study, this correlation seems to be mainly reflected in the male AR children, not significant in female children.

The linkage between AR and ADHD may have a different mechanism: allergic has an effect in increasing activity of TH2 cells and the secretion of the anti-inflammatory cytokines IL-4, IL-5, IL-9,IL-10 and IL-13. TH2-derived cytokines play an important role in the inflammatory process inducing the production of allergen-specific IgE production (IL-4), influx of eosinophils into the into the inflammatory sites of allergic tissue (IL-5) [32]. Inflammatory cytokines can activate neuroimmune mechanisms that involve behaviorally and emotionally relevant brain circuits in animals [33] and humans [34], and may influence the neuronal activity of brain structures indirectly by activation of the hypothalamus-pituitary-adrenal axis. Moreover, inflammatory cytokines further lead to altered metabolism of central neurotransmitters such as norepinephrine and dopamine known to be involved in ADHD pathology. Psychological mechanisms of AR-ADHD comorbidity may be associated with an early exposure to stressful life in children. Stress in life can interfere with the development of ADHD- relevant brain structures, affecting cognitive impairment in children, and leading to symptoms of ADHD or ADHD-like.

Sleep problems are common in children with allergic rhinitis. Previous study revealed that the onset of upper airway inflammation due to allergic triggers in subjects under three years of age may be related to the subsequent development of SDB after 8–10 years [35] . Minor sleep restriction can lead to dysfunction of the circuits in specific regions of the brain, especially the prefrontal cortex, and negatively affect cognitive function and behavior [36] .Impaired executive function is common in children with ADHD. Therefore, sleep problems may play an important synergistic role in increasing the risk of ADHD in allergic diseases. There was a viewpoint that allergic diseases and ADHD have a common genetic mechanism. Signal transducers and activators of tranion6 (STAT6) are involved in the regulation of the immune system, cell proliferation and apoptosis, which thought to play a major role in the pathogenesis of ADHD. Previous study showed that it was closely related to allergic diseases [37]. Moreover, AR may affects the quality of sleep and causes daytime fatigue, cognitive, memory deficit and the learning process [38]. Sleep disorder also could lead to cognitive impairment due to the increase of oxidative stress [39].

This study has some limitations as different parental education level may affect their judgments when filling out the questionnaire, which may cause bias data. Besides, our study is a cross-sectional survey that did not cover the whole range of ADHD patients to establish a causal relationship. Further follow-up of this cohort is warranted to elucidate causal associations.

## Conclusions

This study demonstrated that attention deficits in children with AR are associated with symptoms severity, duration and skin index, especially in male children. The mechanism of the relationship between AR and ADHD symptoms has not been clarified and needs further studies. Interventions incorporating strategies that focus on allergic disease management and collaborative care for children with ADHD deserve further investigation.

## Additional file

Additional file 1: **Table S1**. The IHS question-items, derived from DSM-IV category A symptoms of ADHD (DOCX 13 kb)

## Data Availability

All included.

## Abbreviations

ADHD: Attention-deficit/hyperactivity disorder
AR: Allergic rhinitis
HI: Hyperactivity/impulsivity
IA: Inattention
IHS: Inattention/hyperactivity scale
PRQLQ: Pediatric Rhinoconjunctivitis Quality of Life Questionnaire
SNAP-IV: Swanson, Nolan, and Pelham version IV scale
TNSS: Total Nasal Symptoms Score
