# Supplementary material for: Risk factors analysis of attention deficit/hyperactivity disorder and allergic rhinitis in children: a cross-sectional study
Source: Ital J Pediatr. 2019 Aug 13;45:99. doi: 10.1186/s13052-019-0703-1 (PMC6693261; doi:10.1186/s13052-019-0703-1)
Supplement: Supplementary file 1 — Table S1. The IHS question-items, derived from DSM-IV category A symptoms of ADHD (DOCX 13 kb) [file 13052_2019_703_MOESM1_ESM.docx]

| **Table S1.** **The IHS question-items, derived from *DSM-IV* category A symptoms of ADHD.** |
| --- |
| **The IHS question-items, derived from *DSM-IV* category A symptoms of ADHD.**  THIS CHILD OFTEN . . . |
| 1 . . . fails to give close attention to details or makes careless mistakes in schoolwork, work or other activities |
| 2 . . . often has difficulty sustaining attention in tasks or play activities |
| 3 . . . does not seem to listen when spoken to directly |
| 4 . . . does not follow through on instructions and fails to finish schoolwork, chores or duties |
| 5 . . . has difficulty organizing task and activities |
| 6 . . . avoids, dislikes, or is reluctant to engage in tasks or activities that require sustained mental effort (such as homework or schoolwork) |
| 7 . . . loses things necessary for tasks or activities (eg, toys, school assignments, pencils, books or tools) |
| 8 . . . is easily distracted by extraneous stimuli |
| 9 . . . is forgetful in daily activities |
| 10 . . . fidgets with hands or feet or squirms in seat |
| 11 . . . leaves seat in classroom or in other situations in which remaining seated is expected |
| 12 . . . runs about or climbs excessively in situations in which it is inappropriate |
| 13 . . . has difficulty playing or engaging in leisure activities quietly |
| 14 . . . is “on the go” or often acts as if “driven by a motor” |
| 15 . . . talks excessively |
| 16 . . . blurts out answers before questions have been completed |
| 17 . . . has difficulty awaiting his/her turn |
| 18 . . . interrupts or intrudes on others (eg, butts into conversations or games) |
